# On Dental Decay

**Published:** 1860-01

**Authors:** Francis H. Thomson

**Affiliations:** Glasgow.


### ARTICLE XIV.

*On Dental Decay.* By Francis H. Thomson, M. D., Glasgow.

Few questions are more frequently put to the practitioner than those pertaining to dental caries, and it is to be regretted that but a fractional portion of those, in the department devoted to the practice of dental surgery, are able to give satisfactory answers. This is caused partly from the conflicting theories of Hunter, Fox, Bell, and other contemporaneous writers, or from ignorance ; this latter reason, however greatly to be deplored, is much the case amongst the dentists of this and other countries.

The above mentioned writers all concur in the view that caries almost always, if not invariably, proceeds from some undefined inflammation arising in the interior of these organs, and that, therefore, all caries is developed from within, and gradually comes to the surface. Facts bear us out in thinking the very reverse to be the case. Caries, or gangrene, seems to be an improper term to apply to disease in the teeth, as under every circumstance the destruction of these organs is produced, not from any suppurative process, but merely from the abstraction of the phosphates and carbonates, by the change which takes place in the saliva from alkalinity to acidity, in various diseases and states of the constitution ; it will, therefore, perhaps, be necessary that the composition of the teeth be noted, as laid down by Berzelius, Rhetzius of Stockholm, Purkinje, Müller, Owen, and many others, in the course of their physiological investigations.

The teeth are composed of enamel, bone, dentine, and a substance which has received various names, such as cortical, crusta petrosa, and denominated cement by Owen. The enamel is almost entirely composed of phosphate and carbonate of lime, with a light proportion of organic matter.

ENAMEL contains :

| | |
|---|---|
| Phosphate of lime, with fluoride of calcium, | 88.5 |
| Carbonate of lime, . . . | 8.0 |
| Phosphate of magnesia, . . | 1 5 |
| Membrane, alkali, and water, . . | 2.0 |

The osseous portion, or dentine, hardly differs from true bone. Berzelius found therein :

DENTINE :

| | |
|---|---|
| Cartilage and vessels, . . . | 28.0 |
| Phosphate of lime, with fluoride of calcium, | 64.3 |
| Corbonate of lime, . . . | 5.3 |
| Phosphate of magnesia, . . | 1.0 |
| Soda, with chloride of sodium, . | 1.1 |

In the cortical was found :

| | |
|---|---|
| Organic matter, . . . | 42.18 |
| Phosphate of lime, . . . . | 53.84 |
| Carbonate of lime, . . . | 3.98 |

Thus showing that enamel is almost entirely destitute of organic matter.

The cortical has even more organic matter than the dentine. It may thus be seen that writers on dental caries have every reason to suppose that disease may be similar in its origin to caries in the bones, seeing that in some kinds of dental caries the result appears to be much the same. But any one in the habit of examining diseased teeth, may observe that the alkaline portions are always abstracted, whereas, in true caries, it is the organized matter which is lost ; and although there may be exception to this regulation, yet, in most cases of caries, the process has been the result of inflammation causing suppuration.

The result of experience tends to show that dental caries is produced by mechanical causes and external influences ; and if such be the case, the reason must be evident why the alkaline portions are always abstracted and the vascular or organized left.

It may be necessary to change the term caries to decay, or simple death of the part, and as all subdivisions should be avoided, decay may be classed under two heads—

SIMPLE AND COMPOUND.

Before going into what is the proximate cause of decay, it may be as well to touch on the most commonly accepted theories promulgated by the writers above named.

"The proximate cause of caries," writes Mr. Fox, "appears to be an inflammation in the bone of the crown of the tooth, which, on account of its peculiar structure, terminates in mortification. The membrane which is contained within the cavity of a tooth is very vascular, and possesses a high degree of nervous sensibility, and inflammation of the membrane is liable to be occasioned by any excitement which produces irregular action; and as the bone of the tooth is very dense, and has little living power a death of some part of it may speedily follow inflammation of the vessels of the membrane which is contained within the cavity. When this membrane becomes inflamed, it separates from the bone, and the death of the tooth is the consequence."*

Mr. Bell differs from Fox, in so far that he makes out the proximate cause of caries to be an inflammation of the external surface of the bone immediately under the enamel. He writes—"The true proximate cause of caries or gangrene is inflammation, and the following seems to me to be the manner in which it takes place:—When from cold, or any other cause, a tooth becomes inflamed, the part which suffers most severely is unable, from its possessing comparatively a small degree of vital power, to recover rom the effects of inflammation, and mortification of the part is the consequence."

* Fox, Nat. Hist. of Teeth, part ii, page 12.

Mr. Hunter has come nearer to the true cause, as, although he says that caries is a disease arising originally within the tooth itself, he evidently had a strong opinion that the different articles containing strong menstrua had an effect in assisting caries. Although he held this opinion, it is evident his theory went no further than the possibility of transitory damage caused by the passage of acid food over the surface of the teeth in its way to the stomach; and on experimenting, he found this action to be so slight that he concludes by leaving the subject in the same state, viz. that caries has its origin in the interior of the teeth.

The teeth, being furnished with a lining membrane, and all of them possessed of the same bony structure, should, according to these views, be all subject to the same symptoms, and no class, therefore, should be more frequently the seat of decay than another ; yet it is a fact, admitted by these authors, that this is not the case, for all speak of external and internal, superficial, lateral, deep seated, and many other subdivisions of decay. Mr. Fox says, that the molars are more subject to caries than the other teeth ; that the incisors of the upper jaw are often affected, but those of the under seldom or never. But were the different arguments used upon this subject to be discussed here, the object of this paper would be departed from—which is to give a short explanation of what appears to the writer to be the true cause of decay.

To elucidate the views adopted, it will be necessary to trace to its true source that morbid process by which the teeth are gradually and insidiously destroyed, and it may be as well to speak of simple decay as the first division of the subject.

Simple decay is that which is found in the molars principally, showing itself on the crowns where the enamel meets, from its points of deposition, four in the under and five in the upper, and discoverable at first by a crucial formation of dark lines—the first indication of the decay visible to the eye.

It may be necessary slightly to speak of the peculiar mode of development observed by nature in the formation of the teeth, differing as they do from the other *bones* of the frame.

Each tooth is derived from a separate pulp, observable as early as the fourth month of gestation. These pulps are highly vascular, having an investing membrane, also supplied with its complement of nerves, veins and arteries. These pulps derive their vessels from the artery which passes along the jaw, and the membranes directly from the gums. The bone of the tooth is formed from the pulp, and the enamel from the investing membrane. The osseous matter is deposited on the extreme points of the pulps from the vessels. In the incisors it begins upon the edges, and in the molars upon the points of the grinding surfaces. These ossific depositions soon extend over the surface, till the whole pulp is invested. The enamel secreted from the investing membrane is deposited on these ossific points, in the shape of a fluid that is at first of the consistence of cream, passing into a chalky substance, soon, however, becoming hard, and seeming to undergo a process of crystallization, for it takes a regular and peculiar form. When fractured, it appears to be composed of a number of small fibres, all of which are arranged to pass from the center to the circumference of each point of deposition. As the fetal formation proceeds, new particles are evolved, gradually enlarging each circle of radiation, till all meet in the center of the grinding surface of the tooth. In the healthy organization becoming agglomerated, and forming a strong covering to the more delicate bone structure below, and much less liable to decomposition. But should the development be interfered with in the fœtus by the casual disturbance of the circulation during the period of gestation, it is invariably seen that the vigor necessary for the healthy exhibition of the bone and enamel, is wanting. Thus generally, from an unhealthy pregnancy, the teeth are found to be imperfect. This may not be observed in childhood, as no great note is taken of the first teeth, but, in the permanent teeth, decay is invariably developed at some period, and generally before puberty, when from the unconfirmed substance of the teeth, and imperfect junction of the enamel plates, acid evolved in different complaints, produced by a delicate organization, and in after life superinduced by an artificial mode of existence, attacks the bone through the imperfect joining of the plates. In such states of constitution, the saliva is more or less acid, and although these acids, found in excess in unhealthy, are also to be discovered in normal saliva, experiment has ascertained that they are destructive to the bone of the tooth. Much acid may also be evolved by the food indulged in, and, although not the primary cause of decay, in all probability much increases the carrying out the destruction of these organs. The saliva, according to M. Donné, is in its normal state purely alkaline, and this conclusion is now followed by most physiologists. The pathological condition of the stomach, induced by an acid state of the saliva, is irritation of the mucous membrane; and he contends that this condition usually induces or is accompanied by acidity of the saliva. Besides giving the result of his experiments in arriving at these conclusions, he has narrated a number of cases illustrative of the corresponding change from acidity to alkalinity as the patient recovered from disease.

It may be proper to give the results of some experiments which have been carried out with regard to the action of acid agents on the teeth, and it will not be difficult to show that in various diseases these same acids are evolved.

*First,* Both animal and vegetable acids act on the bone and enamel of the teeth.

*Second,* Alkalies do not act on the enamel.

*Third,* Salts, whose acids have a stronger affinity for the lime of the teeth than for the bases with which they are combined, are decomposed, the acids acting on the teeth.

*Fourth,* Vegetable substances have no effect upon the teeth till after fermentation takes place, but all of them capable of acetic fermentation act readily after the acid is formed.

*Fifth*, Animal substances act very slowly, and even in a state of putridity are tardy in their action.

*Sixth*, Acetic acid so corroded the enamel of a tooth which was placed in it, that in twenty-four hours all that remained could be easily scraped off with the nail. Citric, mallic, muriatic, sulphuric, and nitric acids soon decompose the teeth (and yet how often are those exhibited in medicine without any precaution being used!)

Having thus mentioned the different acids which have effect on the teeth, it will be easy to show that many of them are evolved in certain diseases impregnating the saliva, and thence acting on the teeth.

Simon, in that part of his learned work on animal chemistry, devoted to the investigation of morbid secretions, says, that "the saliva becomes affected in different morbid conditions of the system, but the nature of the changes that it undergoes has not been hitherto sufficiently studied. Morbid saliva sometimes contains a free acid. This is most commonly lactic, but in some cases acetic is also present. The acid reaction may at once be detected by test paper. While normal saliva communicates a blue tint to red litmus paper, this, on the contrary, reddens blue paper. I have frequently," he continues, "seen the saliva acid in acute rheumatism, and in cases of salivation."

According to M. Donné, the saliva has an acid reaction in all cases of irritation and inflammation of the stomach, in pleuritis, encephalites, intermittent fevers, acute rheumatism, uterine affections, and amenorrhæa. Brugnatilli detected oxalic acid in the saliva of a phthisical patient. The secretion of saliva is sometimes increased to an extraordinary degree, constituting salivation. In such cases the chemical characteristics of the saliva are more or less affected. He detected true acetic acid in the saliva of a patient who had just terminated a course of mercury. The saliva was very viscid, of a yellow color, and possessed of a sickly disagreeable smell. The results of his experiments decidedly prove, that in almost every case where he tested morbid saliva, some acid was found, each more or less having an agency in the decomposition of the teeth. His experiments on morbid gastric juice are most interesting, but too elaborate to be more than hinted at in this paper. He says the increased acidity of the gastric juice usually arises from an excess of those acids which exist in a normal state, viz. muriatic, acetic, and lactic acids. When there is a tendency to the formation of an excess of acid in the gastric juice, it appears to be developed from the food—muriatic principally from animal, acetic and lactic from vegetable, especially such saccharine food as bread, beer, and wine, and the fatty acids from an excessive use of fatty matters. Excessive acidity is frequently observed in scrofula, rickets, associated with disease of the spleen, also in gout and nettle-rash.

Having thus given a slight account of the different acids evolved in the saliva and gastric juice when in a morbid state, and having also asserted that decay of the teeth usually, if not always, is found as the result, it may safely be inferred that the saliva so constituted is a most active agent in their destruction. First it is found that, from a delicate gestation, diseased pulps are exhibited, consequently irregular action in the deposition of the bone and enamel; and, as much delicacy and irritation is usually in such cases evinced after birth, causing a morbid state of the secretions, thus laying the foundation of decay, and interfering with the proper development of the teeth, yet this may be so far arrested by the adoption of peculiar treatment and diet. Although decay of the first teeth may not be very evident till near the time of changing, and not much taken into account, yet the action of the different secretions is even more powerful in them than in the permanent teeth; and minute investigation of the molars, the first of which in the second set appear above the gum about the seventh year, will distinctly show the irregularities of the deposition of the enamel before described; and the slightest attack to which children of that age are always more or less liable, such as scarlatina, measles, &c., by producing peculiarly acid states of the saliva and gastric juice, will much increase the process of decay, not very easily accounted for in many cases, and doubtless observed by all practitioners. This decay may be defined as that which is observable on the crowns of the molars, and that insiduous decomposition which takes place so often at the junction of the enamel and bone portion of the tooth where the gum begins.

Although these views of simple decay may not be entirely original, seeing that various authors have slightly alluded to the effect discernible from the action of the different acids on the bone and enamel, no one has described this as the proximate cause of decay, for all talk of internal inflammation of some kind or another being the cause. Inflammation may be the result, but certainly never the cause of decay, for it must be a practically ascertained fact, that pain never exists in a decayed tooth till the nerve becomes inflamed. Now, if this inflammatory action showed itself before the appearance of decay, severe pain would be the result. This, according to the experience of patient and practitioner, never does take place, and toothache proper is only felt when, from the pressure of the decayed mass upon the nerve and vessels in the interior, disturbance ensues. It cannot be denied that much pain is often felt in the interior of the tooth when no decay is visible, but arising from a totally different cause. When, from cold, sudden shock, or constitutional change, inflammation is produced in the interior of the tooth, the result is not visible decay, but suppuration of the nerve itself, which is relieved by exudation through the fangs, causing gumboil and all its attendant miseries. Gumboil also is often the result of a decayed tooth, but this is when, either from injudicious stopping, or from the pressure and non-removal of decayed matter, the discharge resulting from inflammation cannot evacuate itself into the decayed cavity. As a result of this internal inflammation, various wellknown complaints arise in the mouth, such as exostosis, anchylosis, and caries of the sockets.

The second class of decay may be named compound, and is that which is found between all the teeth, but more especially in the incisors and bicuspids, and denominated lateral decay, by many writers. It appears at first as a mere point, gradually increasing till absolute decomposition ensues. It is in treating this part of the subject that a distinct knowledge of the vascularity of the teeth becomes of such importance in the elucidation of a correct diagnosis of this disease. Compound decay would appear to derive its origin from stoppage of the circulation, caused by too close contact of the teeth; for no isolated tooth is ever primarily attacked, or teeth where the enamel has become developed before contact. Some may say the enamel is not vascular, and therefore no damage can take place from contact; but lateral decay never does show itself, except where, from deficiency of the enamel, the bone of the tooth is exposed or very imperfectly covered, and the tooth in contact with its neighbor. In tracing this class of decay, it should be borne in mind, that, in nine cases out of ten, compound decay is developed before puberty, when the teeth are very highly organized, and consequently more likely to suffer from any stoppage of the circulation.

Compound decay may take place at any age, but this is caused by the action of morbid saliva carrying on what may have commenced in infancy, arrested by a strong and healthy boyhood, and again becoming developed by an attack of fever, consumption, or even salivation. The object in making use of the term compound is, that after the morbid condition of the teeth, caused by impeded circulation, has taken place, it may be much increased by the action of the saliva and gastric juice in their morbid states. Hunter observed, that, when caries is communicated by contact, it probably is caused by the action of some acrimonious discharge from the decaying tooth, which, in the first place, occasions a decomposition of enamel, and afterwards of the organs themselves; but there is this peculiar difference, that in the one, decay proceeds from the interior to the exterior, whilst the other commences on the surface. What is there in this to lead one to the true cause? He has, on the one hand, left the proximate cause of true decay much in doubt, and, on the other, only hinted at the action of some discharge, the specific nature of which he does not define. It must be well known to all versant with this subject, that no discharge ever does appear but such as is produced by inflammation going into suppuration, and his theory is rendered still more fallacious by his assertion, that decay must be instituted before any discharge can take place.

From the preceding remarks it must be obvious, that all those who practice dental surgery ought to be acquainted with every department of medical science. A mere mechanical practitioner can never, in the long run, be useful. The more the medical and surgical knowledge of the dentist, the more will he be enabled to alleviate pain or suffering; and the better a man is acquainted with the whole of the healing art, the more successfully will he devote himself to any department to which he may direct his energies. Hitherto, with few exceptions, this branch has been the refuge of those who could succeed in no other, or, what is worse, has much fallen into the hands of uneducated or partially educated adventurers, who, having acquired a few of the manipulations and some knowledge of the mechanical part, have, by bold effrontery and unscrupulous statements, imposed on the unthinking portion of the public. As knowledge advances, the less will be the success of the uneducated and designing; and all who may be, each in his own department, lending a hand to put down quackery in all its branches, are diffusing such a knowledge of natural phenomena, that men, knowing and obeying the Creator's laws, will enjoy the best health, mental and physical, consistent with our present imperfect state of existence. [*Glasgow Med. Jour.*

